# Supplementary material for: Compressive sensing based secure data aggregation scheme for IoT based WSN applications
Source: PLoS One. 2021 Dec 16;16(12):e0260634. doi: 10.1371/journal.pone.0260634 (PMC8675701; doi:10.1371/journal.pone.0260634)
Supplement: Attachment — Submitted filename: report.pdf [file pone.0260634.s001.pdf]

Compressive sensing based secure data aggregation scheme for IoT enabled WSNs applications

Revised title: Compressive sensing based secure data aggregation scheme for IoT Based WSN applications

Reviewers' comments

Reviewer #1	
**Question**	**Answer**
The picture quality of the fig.3 is very poor.	Fixed, the figures are redrawn with better quality
The authors should redraw the figure properly.	Fixed, the figures are redrawn with better quality
The novelty of the work is moderate.	We have revised the paper to highlight the significance of the proposed scheme and create a separate subsection called Motivation and Contribution please see section 1.1
The authors should give the brief explanation about the IoT enabled WSNs in the introduction.	The paper is revised for better understanding and the details about IoT enabled WSNs are provided in introduction: lines 5-12
Why the authors keep the title as IoT enabled WSNs?	Fixed, the title is now, and we changed it into IoT based WSNs instead of IoT enabled WSNs revised
The authors should make formal security analysis.	Thanks for the valuable comments, we have modified the security analysis section accordingly. Please see section 5.1

Reviewer #1	
**Question**	**Answer**
This paper has not clearly shown the advantages in performance of their approach with respect to others from the literature in this field.	Fixed the Related Work section is revised to highlight the benefits of our scheme. Moreover, a formal security analysis is added see section 5.1 to give .bit understanding on the security feature of the proposed work

Indeed, I found the paper a little bit difficult to read, due not only to the poor grammar used throughout, but also the unclear structure of the argument being put across.	Fixed, we have done a major revision of the language as well as the flow of the paper for better readability and understanding
In particular, the quality of the presentation should be improved in this paper.	Fixed, we have done a major revision of the language as well as the flow of the paper for better readability and understanding
This paper would be substantially improved by thoroughly rewriting the prose with the help of a good English-language writer.	Fixed, we have done a major revision of the language as well as the flow of the paper for better readability and understanding
In general, this paper needs such a treatment before being considered any further.	Fixed, we have done a major revision of the language as well as the flow of the paper for better readability and understanding
Furthermore, presentation aside, by reading the paper, it still was not entirely clear what to expect with the direction of the article.	Fixed, the paper is revised for better readability. Moreover, the significance of the proposed work are highlighted in section 1.1
Indeed, the contribution proposed in this paper should properly be compared and contextualized with respect to state of the art.	Fixed the Related Work section is revised to highlight the benefits of our scheme. Moreover, a formal security analysis is added see section 5.1 to give .bit understanding on the security feature of the proposed work
The aspects mentioned above should be carefully addressed before the paper can be considered any further.	Fixed
**Please consider the following remarks to improve your article:**	
Explain novelty of your work presented in this work.	We have revised the paper to highlight the significance of the proposed scheme and create a separate subsection called Motivation and Contribution please see section 1.1
Following are some of relevant and recent references which need to be :discussed in the revised manuscript:<br>1-IoT-based Big Data secure management in the Fog over a 6G Wireless network<br>2- Using Clustering for Forensics Analysis on Internet of Things<br>3- A multi-agent-based data collection and aggregation model for fog-enabled cloud monitoring<br>4- Security in Internet of Things: issues, challenges, taxonomy, and Architecture<br>5- A Secure Decentralized Spatial Crowdsourcing Scheme for 6G-Enabled Network in Box<br>6- IoT transaction processing through cooperative concurrency control on fog–cloud computing environment	Thanks for the valuable comments, we have added the mentioned references please check the related work section.

Paper needs to polish and provide a detailed explication of theoretical aspects such as conditions and theorems, and practical issues like algorithms, rules and possible applications.	Fixed, we have done a major revision of the language as well as the flow of the paper for better readability and understanding
Introduction section needs to be re-written to improve its quality and readability.	Fixed, this section is now revised to improve the readability and understanding
Improve the quality of figures and explain those properly.	Fixed, the figures are redrawn with better quality
**Following are some of relevant and recent references which need to be discussed in the revised manuscript:**	
Many references are with incomplete bibliographic information (like lack of publication venue, for instance). This must be corrected. There are many English and grammatical issues in the paper which needs to be rectified.	Fixed, we have done a major revision of the language as well as the flow of the paper for better readability and understanding
The formula character format is best to be different from the main text, and mathematical characters are recommended.<br>In the related works, "et al" should be "et al.".	Fixed
It seems that the contribution points of the article are a little bit few. After or in the section of Motivation, it is recommended that the authors summarize the contribution points of their work, which clearly demonstrate the innovations.	We have revised the paper to highlight the significance of the proposed scheme and create a separate subsection called Motivation and Contribution please see section 1.1
