# Supplementary material for: Compressive sensing based secure data aggregation scheme for IoT based WSN applications
Source: PLoS One. 2021 Dec 16;16(12):e0260634. doi: 10.1371/journal.pone.0260634 (PMC8675701; doi:10.1371/journal.pone.0260634)
Supplement: Attachment — Submitted filename: Reviewers respond.docx [file pone.0260634.s002.docx]

**Compressive sensing based secure data aggregation scheme for IoT Based WSN applications**

Reviewers' comments

| Editor Comments | |
| --- | --- |
| Question | **Answer** |
| The authors should give more stress for novelty part. | We have revised the paper to highlight the significance of the proposed scheme, please see section 1.1 |
|  |  |
| Reviewer #1 | |
| Question | **Answer** |
| The following papers should be discussed in the related work section properly.  1.Comprehensive survey on security services in vehicular ad-hoc networks, IET Intelligent Transport Systems. 2. Dual authentication and key management techniques for secure data transmission in vehicular ad hoc networks 3.EAAP: Efficient anonymous authentication with conditional privacy-preserving scheme for vehicular ad hoc networks 4.Computationally efficient privacy preserving authentication and key distribution techniques for vehicular ad hoc networks 5.An Anonymous Batch Authentication and Key Exchange Protocols for 6G Enabled VANETs 6.BBAAS: Blockchain-Based Anonymous Authentication Scheme for Providing Secure Communication in VANETs 7.EMBA: An efficient anonymous mutual and batch authentication schemes for vanets. | Thanks a lot for the suggestion, Fixed the Related Work section is revised and the proposed papers have been added and discussed. |
